# Supplementary material for: Applications and outcomes of implementing telemedicine for hypertension management in COVID-19 pandemic: A systematic review
Source: PLoS One. 2024 Aug 1;19(8):e0306347. doi: 10.1371/journal.pone.0306347 (PMC11293715; doi:10.1371/journal.pone.0306347)
Supplement: S1 Appendix — (DOCX) [file pone.0306347.s002.docx]

**Table: Search Strategy**

| Database | Concept 1: hypertension | Concept 2: Telemedicine | Concept 3: COVID 19 |
| --- | --- | --- | --- |
| MEDLINE | 1. Blood Pressure, High  2. Blood Pressures, High  3. High Blood Pressure  4. High Blood Pressures  5. hypertension | 1. Telehealth  2. mHealth  3. Virtual Medicine  4. Mobile Health  5. Telemedicine | 1. COVID 19  2. SARS CoV 2 Infection  3. 2019 Novel Coronavirus Infection  4. 2019 Novel Coronavirus Disease |
| Scopus | 1. Blood Pressure, High  2. Blood Pressures, High  3. High Blood Pressure  4. High Blood Pressures  5. hypertension | 1. Telehealth  2. mHealth  3. Virtual Medicine  4. Mobile Health  5. Telemedicine | 1. COVID 19  2. SARS CoV 2 Infection  3. 2019 Novel Coronavirus Infection  4. 2019 Novel Coronavirus Disease |
| Web of science | 1. Blood Pressure, High  2. Blood Pressures, High  3. High Blood Pressure  4. High Blood Pressures  5. hypertension | 1. Telehealth  2. mHealth  3. Virtual Medicine  4. Mobile Health  5. Telemedicine | 1. COVID 19  2. SARS CoV 2 Infection  3. 2019 Novel Coronavirus Infection  4. 2019 Novel Coronavirus Disease |
| The Cochrane Library | 1.blood pressure  2. hypertension  3.high blood pressure  4. high blood pressures | 1. telemedicine  2. telehealth  3. Virtual medicine  4. Mobile health | 1. COVID 19  2. SARS‐CoV‐2 3. SARS‐CoV‐2 infections |
