# Supplementary material for: Applications and outcomes of implementing telemedicine for hypertension management in COVID-19 pandemic: A systematic review
Source: PLoS One. 2024 Aug 1;19(8):e0306347. doi: 10.1371/journal.pone.0306347 (PMC11293715; doi:10.1371/journal.pone.0306347)
Supplement: S2 Appendix — (DOCX) [file pone.0306347.s003.docx]

**CASP Cohort Study Standard Checklist**

1. Did the study address a clearly focused issue?
2. Was the cohort recruited in an acceptable way?
3. Was the exposure accurately measured to minimise bias?
4. Was the outcome accurately measured to minimise bias?
5. (a) Have the authors identified all important confounding factors?
6. (b) Have they taken account of the confounding factors in the design and/or analysis?
7. (a) Was the follow up of subjects complete enough?
8. (b) Was the follow up of subjects long enough?
9. How precise are the results?
10. Do you believe the results?
11. Can the results be applied to the local population?
12. Do the results of this study fit with other available evidence?
13. What are the implications of this study for practice?
14. What are the results of this study?

| Row | Name/year | Q1 | Q2 | Q3 | Q4 | Q5a | Q5b | Q6a | Q6b | Q7 | Q8 | Q9 | Q10 | Q11 | Q12 | score |
| --- | --- | --- | --- | --- | --- | --- | --- | --- | --- | --- | --- | --- | --- | --- | --- | --- |
| 1 | Alexander  2020 | YES | YES | YES | YES | YES | YES | YES | - | 95% | YES | - | YES | Thus, the COVID-19 pandemic has been  associated with changes in the structure of primary care, with the content of telemedicine visits  differing from that of office-based encounters | Evaluations of cardiovascular  risk factors such as blood pressure and cholesterol have decreased, owing to fewer total visits and  less frequent assessment during telemedicine encounters | 12 |
|  | Alsaqer 2022 | YES | YES | YES | YES | YES | YES | YES | YES | 95% | YES | YES | YES | The intervention group had a significant decrease in systolic blood pressure compared to the control group, but no significant difference was observed in diastolic blood pressure. | A combination of technical and nursing interventions can be effective for improving the quality of life and blood pressure self-care, leading to a statistically and clinically significant reduction in blood pressure among the elderly with hypertension compared to the use of technology. | 14 |
| 2 | Armitage  2022 | YES | YES | YES | YES | YES | YES | YES | YES | 95% | YES | NO | YES | The COVID-19 pandemic has presented an opportunity for  reconfiguring services, in this case to deliver a more accessible  service for patients and one that is resilient to disruptions  in usual care. Looking to the future, ABPM could be one  service that is digitally enabled in both primary and secondary  care. | We have demonstrated that ABPM can be safely and appropriately  provided in the community remotely and without face-to-face  contact, using video technology for remote fitting appointments,  alongside courier services for delivery of equipment to  participants. | 13 |
| 3 | Barake  2022 | YES | YES | YES | YES | YES | YES | YES | YES | - | YES | YES | YES | Further implementation of the HEARTS Initiative in each  center and its expansion to all health centers of the country,  will certainly contribute to recuperate performance indicators  achieved in 2017-2019, and from there on, increase and attain  higher and acceptable control rates that reduce cardiovascular  risk. | All health services decreased their control rates during the pandemic | 13 |
| 4 | Blackstone  2022 | YES | NO | YES | YES | YES | YES | - | - | 95% | YES | - | YES | This information can guide health systems and public health  institutions in developing and expanding telemedicine for  refugee patients. | The results  suggest, while use of telemedicine is significantly lower in  refugee patients compared to non-refugee patients, and there  are barriers to overcome with uptake of telemedicine in refu-  gee patient populations, patients with chronic diseases may  be accepting of telemedicine | 10 |
| 5 | Bruns  2022 | YES | - | YES | YES | YES | YES | YES | YES | - | YES | NO | YES | Telepharmacy visits had a nonsignificant change in BP  control when compared to in-person visits. | VVs were feasible , presented high acceptability , being this modality a valuable tool that complements inperson care | 11 |
| 6 | Burgos  2020 | YES | NO | YES | YES | NO | NO | YES | YES | 95% | YES | - | YES | VVs were feasible, presented  high acceptability, and the overall experience was positive in patients with HF, PH and HT,  being this modality a valuable tool that complements in-person care. | VVs were feasible , presented high acceptability , being this modality a valuable tool that complements inperson care | 10 |
| 7 | Gallardo-Rincón  2022 | YES | YES | YES | YES | YES | YES | YES | YES | 95% | YES | YES | YES | MIDO COVID can help take measures to prevent infection as well as control non-communicable diseases in infected people | MIDO COVID facilitates the planning, surveillance, testing and clinical management of SARS-CoV-2 infections and major non-communicable diseases and their pre-disease states to streamline the continuum of care. | 14 |
| 8 | Gibson  2023 | YES | YES | YES | YES | YES | YES | YES | YES | %95 | YES | YES | YES | Instruction-based multicomponent digital cardiac rehabilitation programs can be successfully implemented and achieve measurable improvements in medical and lifestyle risk factors and psychosocial health. | Interdisciplinary digital cardiac rehabilitation programs can be successfully implemented and help patients achieve the lifestyle, medical, and treatment goals recommended in the guidelines. | 14 |
| 9 | Girerd  2022 | YES | NO | YES | NO | NO | NO | YES | YES | - | YES | NO | YES | The present study shows a gradual decrease in SBP and DBP during quarantine. | Among the very large cohorts, a significant decrease in home BP measured with electronic health devices was observed during the first quarantine period. | 8 |
| 10 | Hernández-Galdamez,  2021 | YES | NO | YES | YES | NO | YES | YES | YES | - | YES | NO | - | In Guatemala, especially in rural settings, access to antihypertensive drugs and health services during the epidemic was disrupted and lower than expected, even after accounting for program activities and implementation measures. | . Telephone calls can provide significant information for monitoring hypertension care and implementation research | 9 |
| 11 | Iliuta  2022 | YES | - | YES | YES | YES | YES | YES | - | - | YES | YES | - | There were no significant changes in blood pressure (BP), heart rate (FC), weight and symptoms or an increase in drug complications between the two periods. | Pandemic restrictions significantly reduced health care utilization, but no significant change in the clinical status of DCM patients under multiparametric home monitoring was observed. | 11 |
| 12 | Lee  2023 | YES | YES | YES | YES | YES | YES | YES | YES | %95 | YES | YES | YES | A teleclinical management program was successfully adapted and provided significant improvements in BP control and increased home BP monitoring despite nationally observed disruption in traditional hypertension care. | Clinical telemanagement programs have the potential to change blood pressure management and care delivery. | 14 |
| 13 | Li, X. Y.  2022 | YES | YES | YES | YES | YES | YES | - | NO | %95 | YES | - | YES | The primary outcome was reduction in systolic and diastolic blood pressure and reduction in heart rate from baseline to the 6-month follow-up visit, proportion of achieving target blood pressure, overall adherence to prescribed medications, and a composite of non-fatal and non-fatal stroke.  Secondary outcomes were adherence and continuation of antihypertensive medications, antiplatelet medications, lipid-lowering agents, proton pump inhibitor (PPI), and antiarrhythmic medications and the cumulative incidence of any cardiovascular-cerebrovascular event. | Telemedicine medication administration for blood pressure management has resulted in better blood pressure control and improved medication adherence than UC during the COVID-19 epidemic, leading to an overall reduction in the incidence of adverse cardiovascular events. | 11 |
| 14 | Moreira  2021 | YES | NO | - | YES | NO | NO | YES | YES | %95 | YES | NO | YES | 13% of patients stated fear of nosocomial infection as the main reason for not attending a pre-scheduled medical appointment. | Telemedicine in cardiology was highly feasible, highly effective, and widely accepted by patients in response to the COVID-19 pandemic, enabling priority case screening and managing outpatient return appointments. | 9 |
| 15 | Nacak  2023 | YES | YES | - | YES | NO | NO | YES | YES | %95 | YES | NO | YES | With remote follow-up, the number of treatment steps has decreased significantly compared to the period before the epidemic (p < 0.05). It was found that 88% of the participants were able to access health services without going to the hospital. | T2DM patients followed with mHealth technologies achieved the necessary metabolic control and treatment compliance during the epidemic. | 10 |
| 16 | Omboni  2021 | YES | YES | - | YES | YES | YES | YES | YES | %95 | YES | YES | YES | There was no significant difference in the proportion of patients treated with antihypertensive drugs during quarantine and after | The results of this study support the usefulness of a telehealth solution for detecting worsening health status during the COVID-19 pandemic. | 13 |
| 17 | Park,  2022 | YES | YES | YES | YES | YES | YES | YES | YES | %95 | YES | NO | YES | The results show that interventions using mHealth can reduce the risk of metabolic syndrome through lifestyle improvements. | Video-based telehealth had positive effects on workers' metabolic risk factors, lifestyle, and service satisfaction. Interventions such as telemonitoring and video counseling using mobile PHR apps are appropriate for workers with poor access to health care during the COVID-19 pandemic. | 13 |
| 18 | Ploux  2021 | YES | YES | YES | YES | YES | YES | NO | NO | - | YES | NO | - | The lockdown was not associated with significant changes in various parameters, including SBP | Quarantine restrictions caused a significant reduction in health care utilization, but did not significantly change the clinical status of HF patients under multiparametric telemonitoring. | 9 |
| 19 | Russo  2022 | YES | YES | - | - | YES | YES | - | - | %95 | YES | - | YES | Remotely treated patients showed lower HbA1c levels | During the COVID-19 pandemic, telemedicine provided an acceptable quality of diabetes care,  comparable to that of patients attending face-to-face consultation, although a less frequent screening of com-  plications seems to have occurred in subjects consulted by telemedicine. | 9 |
| 20 | Sreedhara  2022 | YES | YES | - | - | NO | NO | YES | YES | - | YES | YES | - | BP assessment was significantly less common among telemedicine encounters compared with in-person | Qualitative findings from ARcare and Terros show that telehealth provides consistent or improved health care for some patients with high blood pressure during the COVID-19 pandemic, but not for patients who lack technology or the Internet. , and this shows that telehealth is not a one-size-fits-all solution and needs to be adjusted. | 8 |
| 21 | Steiner  2023 | YES | YES | YES | YES | - | - | YES | YES | %95 | YES | NO | - | In-person primary care visits decreased from 2.7 (2.7) to 1.4 (1.9) per year and virtual contacts increased from 9.5 (12.2) to 11.2 (14.2) per year | that the majority of hypertensive patients who remained under care in an integrated delivery system were able to maintain medication adherence and control hypertension during the first year of the COVID-19 pandemic | 10 |
| 22 | Taylor  2022 | YES | YES | YES | YES | YES | YES | YES | YES | - | YES | YES | YES | these results suggest that video telemedicine platforms offer an efficient, effective, and patient centered approach to address important gaps in hypertension management, including access to care and patient engagement | A holistic approach to blood pressure management based on lifestyle changes and appropriate medication use through virtual primary care video visits is effective across the country. | 13 |
| 23 | Tierney  2023 | YES | YES | - | YES | YES | YES | YES | YES | %95 | YES | YES | YES | Telehealth accounted for 0.33% of encounters in 2019 and increased to 9.55% in 2020 | Higher care continuity is associated with telehealth  use and A1c testing, and lower A1c and blood pressure. Telehealth  use mediates the association of care continuity and A1c testing. Care  continuity may facilitate telehealth use and resilient performance on  process measures | 13 |
| 24 | Walker  262021 | YES | NO | - | YES | YES | YES | YES | YES | - | YES | NO | YES | All participants reported using Zoom to access the program as acceptable, with 83% reporting that it worked well. | A multicomponent weight loss and health promotion program with a low-carbohydrate diet component clinically and statistically significantly improved health outcomes including weight status, blood pressure, and mental health in a cohort of primary care patients at delivery from Remotely improve | 10 |
| 25 | Ye, S27. Anstey 2022 | YES | YES | YES | YES | - | - | YES | YES | 95% | - | YES | N0 | Primary care and telemedicine visits of the heart are not different compared to face-to-face visits | Increased use of telemedicine visits is associated with poorer performance in measuring the quality of hypertension control. However, the use of telemedicine visits may not have a negative impact on blood pressure control during BP recording | 10 |

**CASP Randomised Controlled Trial Standard Checklist**

1. Did the study address a clearly focused research question?
2. Was the assignment of participants to interventions randomized?
3. Were all participants who entered the study accounted for at its conclusion?
4. Were the participants ‘blind’ to intervention they were given?
5. Were the investigators ‘blind’ to the intervention they were giving to participants?
6. Were the people assessing/analyzing outcome/s ‘blinded’?
7. Were the study groups similar at the start of the randomised controlled trial?
8. Apart from the experimental intervention, did each study group receive the same level of care (that is, were they treated equally)?
9. Were the effects of intervention reported comprehensively?
10. Was the precision of the estimate of the intervention or treatment effect reported?
11. Do the benefits of the experimental intervention outweigh the harms and costs?
12. Can the results be applied to your local population/in your context?
13. Would the experimental intervention provide greater value to the people in your care than any of the existing interventions?

| Row | Name/year | Q1 | Q2 | Q3 | Q4 | Q5 | Q6 | Q7 | Q8 | Q9 | Q10 | Q11 | Q12 | Q13 | score |
| --- | --- | --- | --- | --- | --- | --- | --- | --- | --- | --- | --- | --- | --- | --- | --- |
| 1 | Alsaqer 2022 | YES | YES | NO | NO | YES | YES | YES | - | YES | YES | YES | YES | - | 9 |
| 2 | Franco 2022 | YES | YES | NO | NO | NO | NO | YES | NO | YES | YES | YES | - | - | 6 |
| 3 | Kang2021 | YES | YES | NO | NO | - | - | YES | YES | YES | YES | YES | YES | - | 8 |
