# Supplementary material for: Applications and outcomes of implementing telemedicine for hypertension management in COVID-19 pandemic: A systematic review
Source: PLoS One. 2024 Aug 1;19(8):e0306347. doi: 10.1371/journal.pone.0306347 (PMC11293715; doi:10.1371/journal.pone.0306347)
Supplement: Attachment — Submitted filename: Response to Reviewers.docx [file pone.0306347.s004.docx]

Response to Reviewers:

The authors are grateful for the constructive comments of the academic editor and have made the necessary corrections by referring to the available sources, including the PRISMA 2020 checklist, as well as the authors' guideline and articles of the PLOS ONE journal. The following section provides a detailed explanation of each comment and the corrections made based on it.

**Academic Editor comment:** Overall use of English. Please ensure that you are using academic language; do not use conversational phrasing such as "dealt with" or "which stands for". I think this manuscript would greatly benefit from edits from an English native author, or from a language service. Please DO NOT edit English using AI-powered services, such as ChatGPT.

**Authors response:** While we appreciate this constructive comment, we completely edited the article using two language services available to us. We used Google Translate only for the basic translation from Persian to English and its basic editing. Its final and native editing was done by these two language services. We announce that we welcome all suggestions for editing the article by the academic editor and respected referees.

**Academic Editor comment:** Please make sure that your spelling and abbreviations are consistent (such as spelling COVID-19 correctly, and not "Covid 19".

**Authors response:** Thanks to this constructive comment, the necessary corrections have been made.

**Academic Editor comment:** Make sure all of your sentences and paragraphs are complete (such as incomplete sentence on line 133).

**Authors response:** The manuscript was subjected to a meticulous examination, during which the necessary corrections were implemented.

**Academic Editor comment:** The search strategies on appendix 1 is woefully insufficient. Apart from significant spelling errors (such as "Cochran", and "Web of Since"), make sure that a separate table is provided for each database, with **replicable search statements** (i.e., if the statement is entered into the platform, it would replicate the author's search exactly). At the moment, I have no idea what exact strategy the authors used.

**Authors response:** In light of the comment, the search strategy was entirely revised.

**Academic Editor comment:**  Authors also need to review their systematic review methodology knowledge and ensure that PRISMA 2020 is followed correctly. For instance, the authors used the CASP framework for item 14 on PRISMA 2020. This is incorrect; item 14 is referring to publication bias, and should be evaluated appropriately. Please review the checklist and revise your methodology. Same for item 15. Quality of evidence is different from per-study risk of bias assessments.

**Authors response:** The authors would like to express their profound regret for the fault in the completion of items 14 and 15 of the PRISMA checklist. Item 14 examines publication bias, which is often used in meta-analysis studies, and the Egger test or other statistical methods are often used. Item 15, which addresses the assessment of certainty, is also used in meta-analysis. Since there is no meta-analysis in our study, items 14 and 15 are not applicable (NA). The very small number of RCTs was one of the reasons for not conducting a meta-analysis.

**Academic Editor comment:** The authors did not append a PRISMA flowchart, as required by the PRISMA guideline.

**Authors response:** In the previous version of the manuscript, we presented the PRISMA 2020 flow diagram named Figure 1. To capture more details, we have re-drawn the diagram using Shiny App.
